# A novel SETX gene mutation associated with Juvenile amyotrophic lateral sclerosis

**DOI:** 10.1002/brb3.1066

**Published:** 2018-07-27

**Authors:** Limin Ma, Yingying Shi, Zhongcan Chen, Shujian Li, Jiewen Zhang

**Affiliations:** ^1^ Department of Neurology People's Hospital of Zhengzhou University Zhengzhou China; ^2^ Department of Neurology Henan Provincial People's Hospital Zhengzhou China

**Keywords:** autosomal dominant, juvenile amyotrophic lateral sclerosis, SETX gene

## Abstract

**Objects:**

This study aimed to report a novel point mutation associated with juvenile amyotrophic lateral sclerosis (JALS) in a Chinese Han family.

**Methods:**

Detailed clinical assessment was applied to two patients, including proband (II‐2) and his mother (I‐2). Next‐generation sequencing (NGS), also known as high‐throughput sequencing in whole exon sequence, was performed in the proband to reach the target region. Sanger sequencing was also used to detect DNA sequence variants of the proband and other three members of his family.

**Results:**

The proband (II‐2) and his mother (I‐2) were successfully diagnosed according to the clinical manifestations and physical examination. A novel point mutation c.1157T > C in the exon 10 of the SETX gene was identified in II‐2 and I‐2, resulting in a substitution of methionine (ATG) to threonine (ACG). However, we ultimately did not find the same variant in the other two normal members of his family in addition to 100 unrelated normal subjects.

**Conclusion:**

We presented a novel probably pathogenic missense mutation in exon 10 of SETX gene in a Chinese Han family with JALS.

## 1 INTRODUCTION

Amyotrophic lateral sclerosis (ALS), also known as motor neurone disease (MND) and Lou Gehrig's disease, is a neurodegenerative disease characterized by progressive muscle weakness due to loss of both upper and lower motor neurons (Thompson et al., 2018), and patients eventually died within 2–3 years after the onset of the first symptoms (Nishiyama et al., 2017). It is the most frequent motor neuron disease, and prevalence is five per 100,000 population (Maurel et al., 2018). About 5%–10% of ALS patients have a family medical history, these cases are classified as familial ALS (FALS), whereas the remaining 90%–95% of cases are sporadic ALS (SALS) (Nishiyama et al., 2017). It has been widely accepted that genetic factors play an important role in both FALS and SALS (Liu et al., 2017). Since SOD1 gene was first recognized as a pathogenic gene of FALS in 1993 (Nishiyama et al., 2017), the genetic basis of the disease with the implication of more than 30 genes currently associate with FALS (Maurel et al., 2018).

The typical age of disease onset is more common in people who are in the range of 60–69 years (Recabarren‐Leiva & Alarcon, 2018). Juvenile amyotrophic lateral sclerosis (JALS) is a rare form of ALS with onset prior to age 25 years, which is characterized by slower progression and prolonged survival to a normal life expectancy (Leblond et al., 2016). Most of JALS cases were inherited in an autosomal recessive pattern. Mutations in senataxin (SETX) gene were described as a rare underlying cause of autosomal dominant form of juvenile‐onset FALS (Liu et al., 2017). According to the Human Gene Mutation Database (HGMD, http://www.hgmd.cf.ac.uk/ac/index.php), only 17 SETX mutations have been yet reported to associate with ALS. In this study, we attempted to report a novel pathogenic SETX mutation in a Chinese Han family with JALS.

## 2 MATERIALS AND METHODS

### 2.1 Subjects

**Figure 1:**
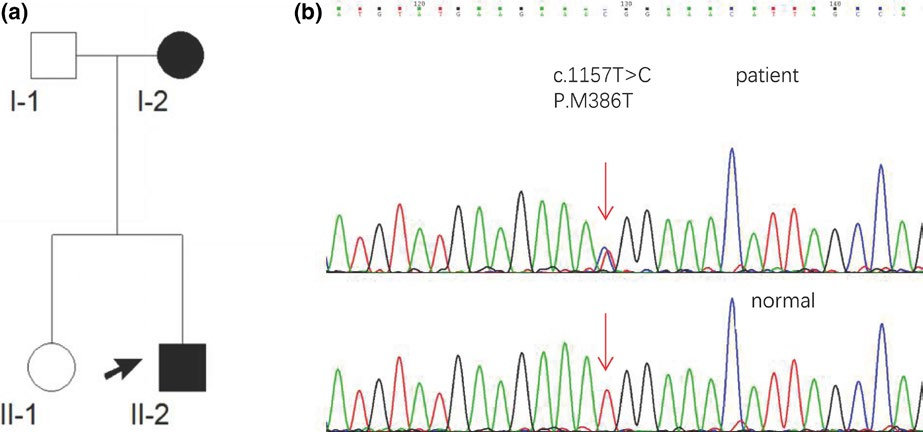
(a) The family tree of the SETX Met386Thr mutation pedigree. The arrow means the proband (II‐2); square, male; circle, female; black symbol, affected family member. (b) DNA sequencing chromatograph of exon 10 of the SETX gene (abnormal and normal), SETX gene with a heterozygous mutation: c.1157T > G (p.Met386Thr)

The pedigree is composed of four family members in two generations, and the family members were enrolled their blood to the Department of Neurology of People's Hospital of Zhengzhou University (Zhengzhou, Henan province, China) (see Figure 1a). The proband was evaluated and clinically diagnosed at the mentioned hospital. The diagnosis of patients with ALS was performed according to the “El Escorial revisited: Revised criteria for the diagnosis of amyotrophic lateral sclerosis” (Brooks, Miller, Swash, & Munsat, 2000). Besides, 100 unrelated normal subjects were recruited as well.

### 2.2 Clinical assessment

The family medical history was obtained from the proband and his parents. The proband underwent magnetic resonance imaging (MRI) of his head and cervical cord, electromyography (EMG) examination, and lumbar puncture. The proband also underwent some laboratory examinations including a complete blood count, the level of thyroid hormone, erythrocyte sedimentation rate, c‐reactive protein, and levels of folic acid vitamin B12. The proband's mother underwent the same laboratory examinations in the hospital.

### 2.3 Analysis of gene mutations

Blood samples from the proband (II‐2) and the other three members of his family (see Figure 1a), and 100 enrolled unrelated normal subjects were collected in EDTA (5 ml) vacutainers, and written informed consent was obtained from all participants as well. Genomic DNA was extracted from peripheral blood leukocytes using standard procedures. All coding exons, splice sites, intron sequence immediately adjacent to alternatively spliced exons high‐throughput DNA sequencing (99.62% of coverage gene and 338.17X of coverage depth) were analyzed for whole exon sequence in the proband (II‐2) to reach the target region. Sanger sequencing method was also used to verify the proband's DNA sequence variants and the other three members of his family. The target‐specific polymerase chain reaction (PCR) primers were designed based on the SETX gene mutation. The PCR involves the primers mediated enzymatic amplification of DNA, which were forward 5′‐TCCGTGTAAACGCAGTGGTAG‐3′ and reverse 5′‐GACTTGGGTGTTGAACCTTGTC‐3′. PCR amplification was performed by MultiGene OptiMax (Labnet International, Inc., NJ, USA) under the following conditions: at 94°C for 30 s, at 55°C for 30 s, and at 72°C for 1 min. After PCR amplification, PCR products of the proband and the other three members of his family were purified and directly sequenced on an ABI3100 automated sequencer (Applied Biosystems, CA, USA). Sequencing reads and lists were compared using Chromas DNA sequencing software and BLAST. The identified single nucleotide polymorphism was determined using the HGMD, dbSNP‐137, HapMap, the 1000 Genomes Project, and the ExAC Browser (Beta). The pathogenicity of the single nucleotide mutation was predicted using PolyPhen‐2, SIFT, and MutationTaster, along with the conservation score. The study protocol was also approved by the Institutional Review Board of People's Hospital of Zhengzhou University.

## 3 RESULTS

### 3.1 Clinical assessment

The proband (II‐2), who was a 11‐year‐old boy, admitted to the hospital at 2016‐07‐18. Approximately 2 years before his admission, he started to complain from distal weakness in the lower limbs, and he felt fatigue after walking at short distances. Gradually, he appeared with an abnormal gait and easily fell down, while he was walking and running. After a while, his symptoms were progressively worsened, and muscle atrophy was observed by his parents as well. On admission, the physical examination showed an abnormal gait, distal lower limb muscle atrophy and fibrillation, contracture of Achilles tendon, decrease in strength of lower limb muscle (the left 3/5, the right 2/5), brisk reflexes of arms and legs, and positive Hoffman's sign, while the strength of the upper limb, the proximal lower limb, intelligence, and sensory were in normal status. The EMG results showed a decrease in the amplitude of compound muscle action potential, as well as fibrillations of tibialis anterior muscle and peroneus; however, nerve conduction velocity (NCV) of the radial nerve (motor and sensory) was normal. The MRI of the head and cervical spine was normal as well, and the result of cerebrospinal fluid (CSF) showed a normal status.

### 3.2 Family medical history

The proband's mother (I‐2), who was 36 years old, had similar symptoms with the proband. At the age of 19 years old, she manifested weakness of distal lower limbs and then appeared the distal weakness in upper limbs at the age of 28 years old. Her symptoms slowly progressed. The physical examination showed the distal weakness in lower limbs, hyperfunction of tendon reflexes, and bilateral cone bundle asks for masculine gender. The EMG performed at a local hospital showed the dysfunction of pyramidal tract and damage to lower motor neurons.

### 3.3 Mutational analysis

Sanger sequencing of the proband's genomic DNA showed a point mutation (c.1157T > C) in the exon 10 of the SETX gene, resulting in a substitution of methionine (ATG) to threonine (ACG) (see Figure 1b). No mutations in other genes were found. Segregation analysis of other three members of family showed that affected subject (I‐2) was an obligate carrier. This missense mutation was not reported in the HGMD and previous studies as well. In addition, the mutation was not a single nucleotide polymorphism (SNP). The mutational analysis using SIFT, Polyphen‐2, Phylop, and MutationTaster resulted in predicted damaging, probably damaging, and conserved and disease‐causing effects, respectively. The other two normal members of family and 100 unrelated normal subjects did not carry the mutation.

## 4 DISCUSSION

In the present study, we have successfully proposed a novel missense mutation in exon 10 of the SETX gene in a Chinese Han family with JALS. The pathologic nature of the Met386Thr mutation was supported by the phylogenetic conservation of the residue. Multiple sequence alignment for SETX was performed in comparison with different organisms, and evolutionary conservation analysis indicated that the novel p.Met386Thr mutation resulted in a change occurred in an amino acid that is highly conserved. In silico prediction of the effects of mutations on the human, the novel mutation was probably damaging with a score of 0.99 obtained by Polyphen‐2. Hence, we speculated that the missense mutation (p.Met386Thr) in our study is probably pathogenic, and it probably has a relationship with the JALS pedigree.

Juvenile‐onset ALS is associated with the great majority of the kindreds, with both autosomal recessive and autosomal dominant pattern of inheritance (Conte et al., 2012), and mutations in several genes such as alsin (ALS2), SETX, spatacsin (SPG11), SOD1 (Orban, Devon, Hayden, & Leavitt, 2007), and FUS (Belzil et al., 2012; Conte et al., 2012; Zou et al., 2013). In Chinese Han families with JALS, FUS mutations were reported to be the most common genetic determinant with an aggressive phenotype (Liu et al., 2017), especially the de novo ones (Zou et al., 2013). Mutations in the SETX gene are associated with a rare and autosomal dominant form of JALS, also known as ALS4, which is characterized by motor neuron dysfunction of early onset, slow progression, acute muscle weakness and pyramidal tract signs, and the absence of bulbar and sensory abnormalities (Orban et al., 2007). According to the literature, 17 missense mutations in SETX gene were reported to be associated with ALS4 (Tripolszki et al., 2017). Except p.N264S and p.T1118I mutations which were associated with atypical phenotypes, other 15 heterozygous missense mutations all had typical phenotype involved by upper and lower neurons (Tripolszki et al., 2017). In the family examined in our study, the two patients all have the typical phenotype of distal lower weakness, pyramidal tract signs, and the absence of sensory abnormalities, which is in accordance with the literature. However, the age of disease onset of the proband is 9 years old, which is younger than his mother whose onset age is 21, suggesting that other factors, such as endogenous, epigenetic, or even environmental factors, may significantly affect the clinical onset age of the disease.

SETX gene encodes DNA/RNA helicase (senataxin protein) consisting of 2677 amino acid residues (Moreira et al., 2004), which are involved in DNA repair, replication, recombination and transcription, RNA processing, transcript stability, and translation initiation (Chen et al., 2004). SETX missense mutations might lead either in heterozygous or homozygous/compound heterozygous condition to ALS4 or Ataxia‐ocular apraxia 2 (AOA2)(Arning et al., 2013), which is characterized by progressive cerebellar ataxia with peripheral neuropathy, cerebellar atrophy, occasional oculomotor apraxia, and elevated alpha‐fetoprotein (AFP) serum level (Arning et al., 2013); however, insertion/deletion of relevant mutations may cause AOA2. It is possible that a toxic loss of function or a partial loss of function in the SETX protein causes a motor neuron degenerative disorder, whereas loss of function of SETX leads to AOA2, a disorder with more widespread pathology (Orban et al., 2007). According to the literature, numerous AOA2‐related missense mutations were described which would be clustered within the N‐terminus and helicase domains (Arning et al., 2013). In the present study, a novel pathogenic ALS4 missense mutation was localized in the N‐terminus of the SETX gene with only two ALS4‐related missense mutations whose have been reported so far. However, no functional mechanism has been delineated to explain the distinct allelic phenotypes and modes of inheritance. In the future, we will attempt to explore the mechanism of the novel mutation through further functional studies.

In conclusion, we presented a novel probably pathogenic missense mutation (p.Met386Thr) in exon 10 of SETX gene in a Chinese Han family with JALS. Although SETX related mutations in JALS is rare, it is still necessary to screen SETX gene in patients with JALS. Our study enriched the gene bank of JALS as well. However, further studies to identify the pathogenic mechanism of the p.Met386Thr mutation should be conducted in the future.

## CONFLICT OF INTEREST

The authors declare that there is no conflict of interest regarding publication of this article.
